# A Quantitative Model of International Trade Based on Deep Neural Network

**DOI:** 10.1155/2022/9811358

**Published:** 2022-05-31

**Authors:** Xiaoxin Huang, Xiuxiu Chen

**Affiliations:** School of Finance and Trade, Wenzhou Business College, Wenzhou, Zhejiang 325035, China

## Abstract

This paper is an in-depth study of international trade quantification models based on deep neural networks. Based on an in-depth analysis of global trade characteristics, a summary of existing problems, and a comparative analysis of various prediction methods, this paper constructs the ARIMA model, BP neural network (BPNN) model, and deep neural network (DNN) model to make a comprehensive comparison of international trade quantification. The results show that the nonlinear model has a global trade quantification has some advantages over linear models, and the deep model shows better prediction performance than the shallow model. In addition, preprocessing of the time series is considered to improve the prediction accuracy or shorten the model training time. The empirical modal analysis method (EMD) is introduced to decompose the time series into eigenmodal functions (IMFs) of different scales. Then the decomposed IMF series are arranged into a matrix using principal component analysis (PCA) to reduce the dimensionality and extract the data containing the most stock index information features; these features are then input into BPNN and DNN for combined prediction, thus constructing the combined models EMD-PCA-BPNN and EMD-PCA-DNN. Based on Melitz's heterogeneous firm trade theory and its development by Chaney, a quantitative trade model incorporating production heterogeneity is constructed through a multisector extension. This paper adopts a general equilibrium analysis, which makes the modeling process pulse clear. The completed model has high flexibility and scalability, which can be applied to quantitative analysis of various problems.

## 1. Introduction

Since the reform and opening up, foreign trade has become a catalyst for economic growth. Foreign trade exports play an irreplaceable role in increasing employment, boosting economic growth, promoting the development of foreign exchange earnings, etc. Between 1978 and 2019, the total import and export volume grew from 35.5 billion yuan 1978 to 31.54 trillion yuan, ranking among the top global trade powers [1]. However, despite the expanding scale of China's trade, exports are still dominated by labor-intensive products, with a relatively low share of exports of technology-intensive and capital-intensive products. Although the proportion of exports of high-tech products has increased in recent years, there is still a more obvious gap compared with developed countries [2]. At the same time, although the trade in services is growing steadily, it is still at a low level in terms of proportion, and the labor-intensive exports are also the main ones. Therefore, what factors will affect the structure of trade goods and how to make the export products develop towards high technology level becomes an important issue.

Counterfactual simulations using quantitative models require only actual observational data, not so-called “natural experiments.” It is difficult to find suitable “natural experiments” for many of the issues related to trade policy that we want to understand. The so-called “quantitative trade model” can be understood as a small-scale Computable General Equilibrium (CGE) model [3]. Compared with the CGE model applied in macroeconomics, the quantitative model based on trade theory is constructed directly based on the trade model. Fewer factors are considered, and only the characteristics of the trade aspect are portrayed. However, quantitative trade models are more advantageous than general CGE models when studying issues related to trade policy because they describe trade characteristics in more detail it has a stable investment performance. It can overcome human weaknesses and achieve sound investment with strong information processing ability [4]. The development and application of quantitative trade models is a relatively new research area, originating from understanding and studying “Gravity Models.” In international trade, “gravity models” are theoretical models that derive the Trade Gravity Equation (TGE). The Trade Gravity Equation is an equation that describes the amount of bilateral trade (bilateral trade flow). Initially, it was widely used as an empirical equation in empirical studies, at least in the 1960s, with great success.

There are several difficulties to overcome in constructing quantitative models, the most obvious one being the treatment of the attribution of profits due to fixed export costs [5]. This paper overcomes these obstacles and demonstrates that the model can be applied to analyzing specific problems, which may be a helpful development and addition to quantitative trade research. Moreover, this paper extends the model to the multisectoral level, which has two advantages: first, the multisectoral model can analyze the impact of trade policy shocks on different sectors, and second, the multisectoral model can be more directly linked to trade data [6]. Second, the idea in constructing the quantitative model in this paper is first to derive the general trade gravity equation describing the bilateral trade volume and then use it as a pivot to analyze the general equilibrium conditions of the model and explicitly write a set of available equilibrium equations, and then directly use the set of general equilibrium equations as the basis for simulation analysis, which is normative and structured [7]. The modeling method in this paper has fewer restrictions on the model and has relatively good scalability. In principle, the modeling approach of this paper can be applied to any quantitative application of the gravitational equation model. Finally, the counterfactual numerical simulations performed using the model in this paper show that the model's quantitative analysis of trade policy issues is predictive and leads to quite detailed analytical results, which is a feature of the quantitative model constructed in this paper.

## 2. Related Works

As early as the 1960s, the famous scholar Park B studied the quantization effect problem [8]. He concluded that if a finite list quantizer quantizes a controller, it may limit and chaos problems in the feedback control system. After that, many scientists have invested in the research of quantization effects on systems [9]. Especially in recent years, the development of communication and network technology and the limitation of network transmission capacity in practice have made quantization control get more and more attention in network control systems, mainly including the influence of quantizers on control systems and quantizer design problems, and many results have appeared accordingly [10]. Considering single-input and multi-input plans, the literature designs pairwise quantizers for the two cases. It obtains sufficient conditions for the stability of the discrete system and the corresponding controller design scheme by constructing Lyapunov generalized functions. By introducing the sector-bound method, the quantized feedback control is transformed into a robust control optimality problem, and the results are extended to multi-input multi-output systems. Also, considering the quantization and event triggering mechanisms, the network transmission capability is improved by setting quantizers in the state feedback channel and the control input channel separately, and the controller is designed [11]. In this paper, we apply this method to replace the controlled object from an available continuous system with a hopping system and study the effect of quantization on the system performance.

With the in-depth study of CNN, it was found that when the number of layers of CNN reached a specific number, the network did not improve its accuracy and led to the degradation of the network performance [12]. Dibia and Demiralp proposed the Residual Neural Network (Res Net) in 2015. Res Net won the ILSVRC that year with a The Res Net network model refers to the ideas of the two network models, Highway Network and Network in Network and adds Short Connection and Bottleneck structures to the network structure [13]. The network deepens to 152 layers to achieve a more profound and broader network for training. This led to a dramatic increase in the number of parameters, so the network Res Ne Xt can improve the accuracy without increasing the complexity of parameters. The Dense Net proposed in 2017 extracted the best part of the Res Net and developed the network potential from feature reuse [14]. Instead of combining features by summation, the elements were connected by a connection. The input of each layer of the network is the concatenation of the outputs of all previous coatings. This way alleviates the gradient disappearance, significantly reducing the number of parameters.

In terms of our traditional international trade settlement, Nyflot et al. point out that letters of credit can have ambiguous terms in the settlement process or even words considered unattainable by the other party, which require extra attention from both sides of our trade [15]. Shi et al. construct a panel statistical data model from the RMB's denomination and settlement function to explore the significant impacts of cross-border trade on RMB settlement according to each region [16]. Yang et al. explore and delve into the concept and possibility of Alipay as a means of international trade settlement through a comparison of the interaction between Alipay and other countries' e-commerce settlement means and methods and a practical case study of its overseas strategies [17]. As one of the primary means of international trade, Zhai et al. pointed out that a letter of credit has always been throughout the change in global settlement management and plays a significant role in the competing process between buyers and sellers [18]. Heo et al. explore the impact of RMB exchange rate fluctuations on inflationary situations and discuss the internationalization of RMB and the role it should play in international trade [19]. Muhammad et al. analyze the use of letters of credit in trade activities and provide a detailed explanation of the meaning, process, and division of letters of credit to provide investors with some concrete ideas on how to use trade tools to avoid trade risks in international trade [20]. Ran et al. comprehensively analyzed the problems in collection, remittance, and letter of credit and the risks caused by using different settlement instruments and provided effective control strategies to reduce the chances of settlement instruments [21]. Khan et al. pointed out that, with the current rapid development of the world market economy and the globalization of trade, market competition is increasingly fierce, in the aggressive global international business and investment competition, global commodity import, and export international trade and investment activities will continue to expose our enterprises to many global risks [22]. The traditional international trade foreign exchange settlement management model still needs to be constantly updated to transform and upgrade to fully meet our urgent needs for the development of international trade. Lundervold and Lundervold discuss the risks of letters of credit in international trade and the countermeasures to prevent them, which effectively guide export enterprises' development of the international trade business [23].

## 3. Design of Quantitative Models for International Trade Based on Deep Neural Networks

### 3.1. Depth Neural Network Model Construction

**Figure 1:**
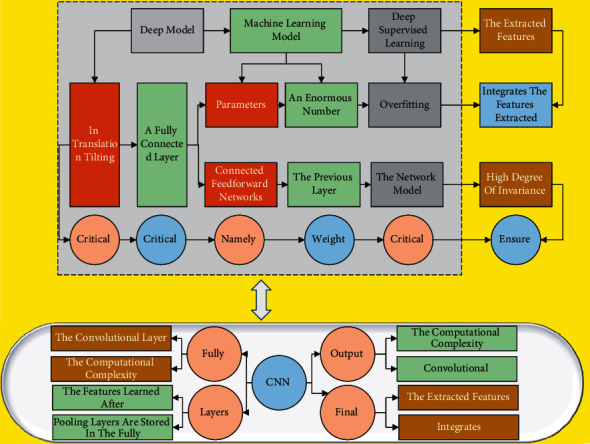
Schematic diagram of the structure of the convolutional neural network.

CNN is a kind of machine learning model under deep supervised learning; as a deep model, CNN model parameters will increase, then it needs a vast data set for training. In the face of such an enormous number of parameters will quickly lead to overfitting. Previously, fully connected feedforward networks had the feature that each neuron is connected to all elements of the previous layer, so it is conceivable that if the network model is trained, it will face many parameters. CNN proposes two critical ideas, namely local connectivity and weight sharing, to optimize the network structure by combining local perceptual regions and shared weights to make full use of the data's local information and ensure a high degree of invariance in translation tilting and scaling. The layer structure generally consists of an input layer, a convolutional layer, a pooling layer, a fully connected layer, and an output layer. The convolutional layer performs convolutional operations to extract graph features. The pooling layer selects the extracted features and integrates the features extracted from the convolutional layer to reduce the computational complexity. The features learned after the convolutional and pooling layers are stored in the fully connected layer. The sample labels are compared with the learned high-level features in the application. The judgment results are sent to the output layer, and then the final results are output. The structure of the convolutional neural network is schematically shown in Figure 1.

#### 3.1.1. Input Layer

Input layer (INPUT), for short, is the input to the data and can-do data preprocessing and can process multidimensional data. Since the gradient descent algorithm is used for learning, the input features need to be normalized, which helps to improve the learning efficiency and performance of the convolutional neural network.

#### 3.1.2. Convolutional Layer

**Figure 2:**
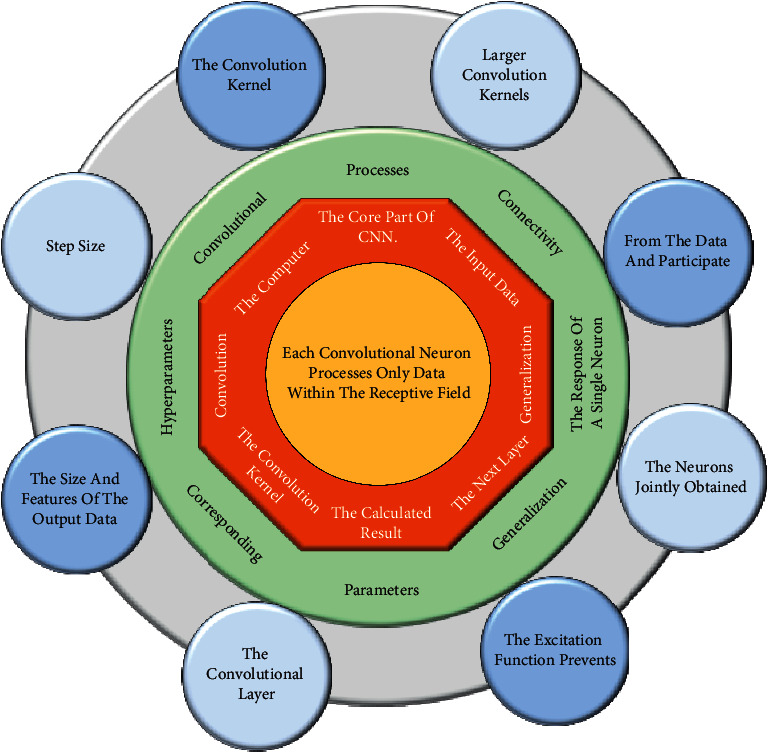
Convolution calculation process.

The convolutional layer (CONV), or CONV for short, is used to extract the feature information of the input data and is the core part of CNN. The image as input data is represented as a numerical matrix in the computer, and the convolution is done in the convolution layer with the convolution kernel. The calculated result is used as the input of the next layer. The convolutional operation is linear in a convolutional neural network, and the data samples are not necessarily linearly divisible. If no activation function is used, then no matter how many layers the CNN has, the output of each layer is a linear combination of the input of the previous layer, so the CNN is still a linear model with limited expressiveness. Convolution simulates the response of a single neuron to a visual stimulus, and each convolutional neuron processes only data within the receptive field, i.e., local connectivity, which significantly reduces the number of parameters and improves the generalization ability of the model. Taking a 5 × 5 input as an example, the size of the convolution kernel is 3 × 3, and the step size is set to 1. The process is that the convolution kernel is sliding over the input feature map one by one, and each slide will compute a result, which is the corresponding pixel value of the output feature map. The convolutional computation process is shown in Figure 2.

The convolutional layer mainly includes the hyperparameters of convolutional kernel size, step size, and padding, which can control the size and features of the output data in the convolutional layer. In contrast, the excitation function prevents the output of the neurons jointly obtained by the connection. Smaller convolutional kernels extract more detailed features from the data and participate in less training to improve the training speed but are less capable of extracting abstract features from images. Larger convolution kernels can better remove abstract elements from the data, but they also involve more computation and increase the training time [24]. The step length (stride) indicates the adjacent distance of the feature map swept before and after the convolution kernel; when the step length is set to 1, the convolution kernel is swept through the feature map one in the convolution layer. Padding is used to solve the problem that the size of the output will become smaller and smaller after the convolution operation and is also used to deal with the problem that the pixel information at the edges may only participate in the bit operation once in the loss of edge information. The following three padding methods are often used: valid padding, same padding, and full padding. The feature map size obtained after the convolution calculation satisfies the following relationship with the size of the convolution kernel, the step size, and the padding.

(1) $$\begin{array}{c} P=\frac{\sqrt{P_{1}-2P+K}}{S-1},\\ H=\frac{\sqrt{H_{1}+K-2P}}{S+1}, \end{array}$$

where *P*2 and *H*2 are the width and height of the output feature map after convolution, respectively, *P*1 and *H*2 are the width and height of the input feature map before the convolution operation, *k* is the size of the corresponding convolution kernel, *P* is the number of ZeroPadding, and *S* is the step size. In the convolutional layer, the activation function is a critical concept. The convolutional operation is linear in a convolutional neural network, and the data samples are not necessarily linearly divisible. If the activation function is not used, then no matter how many layers the CNN has, the output of each layer is a linear combination of the input of the previous layer, which constitutes a CNN is still a linear model with limited expressiveness. The Sigmoid function is a saturation activation function that compresses an arbitrary size input to between 0 and 1. If it is a huge negative number, the more it is compressed, the closer it is to 0. If it is a huge positive number, the more compressed it is, the closer it is to 1. It is often used as a binary classifier in neural networks. It is often used as the activation function in the last layer of a neural network, which can convert any real value into a probability. However, if the Sigmoid activation function is used in a deep neural network, it can cause the problem of gradient disappearance and gradient explosion of the neural network. The formula of its position is

(2) $$\begin{array}{c} \text{Sigmoid}\left(x_{1}\right)=\sum_{e=1}\left(e-x\right). \end{array}$$

Tan*h* activation function also belongs to the saturation activation function; compared to the Sigmoid function, it has a more extensive range of values. For (−1, 1), it is proposed to solve the Sigmoid function is not a zero-centered output problem. The Equation of its operation is

(3) $$\begin{array}{c} \text{Tanh}\left(x_{1}\right)=\sum \frac{e^{x}+e^{-x}}{\sqrt{e^{x}-e^{-x}}}. \end{array}$$

The ReLU function is a frequently used activation function in current deep learning models. It belongs to the unsaturated activation function, which at the mathematical level is the function that takes the maximum value. When the input is negative, it is all set to zero, and when the information is positive, it remains unchanged. This feature is called one-sided suppression. In the hidden layer, this feature brings some sparsity to the output of the hidden layer and improves the computational speed. The Re LU class activation function has a relatively better performance and can significantly improve the training speed of the network without affecting the accuracy. Its role is given by

(4) $$\begin{array}{c} RELU\left(x\right)=\sum \max\sqrt{\left(x+1\right)}. \end{array}$$

#### 3.1.3. Pooling Layer

**Figure 3:**
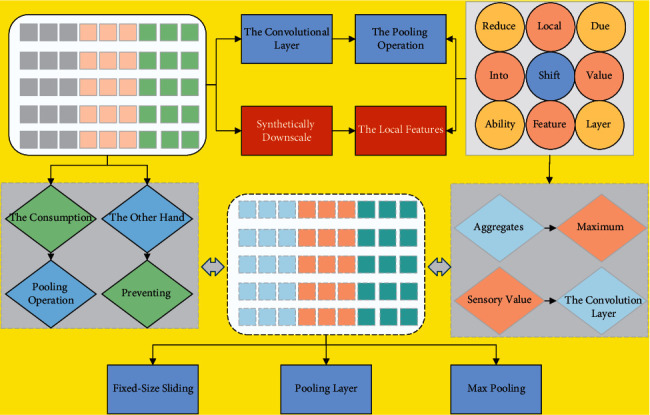
Schematic diagram of maximum pooling and average pooling.

The pooling layer (POOL) is usually placed behind the convolutional layer for operation. On the one hand, the pooling operation can synthetically downscale the local features of the feature map, which means the input image in the next layer will be smaller, thus reducing the network parameters and computation and thus reducing the consumption of computer resources; on the other hand, the pooling operation will select the best features in the feature map, and play a role in preventing overfitting. The process of the pooling layer is to use a fixed-size sliding window that slides over the input image and aggregates the elements in the sliding window into one value at a time as the output. Depending on the aggregation method, it can be divided into maximum pooling and average pooling. Max pooling is to calculate the top point of the output local sensory value, which can reduce the error caused by the mean shift of the estimation due to the mistake of the convolution layer parameters by keeping more texture information during the feature extraction. Average pooling calculates the mean of all values in the output local perceptual field. It reduces the error caused by the increase in the estimate variance due to the limitation of the neighborhood size during feature extraction by retaining more information about the image background. The two pooling operations are shown in Figure 3. The top pooling layer has the advantage of sparsity over the average pooling layer, conducive to improving the model's generalization ability. In practical applications, the maximum pooling method is often used.

#### 3.1.4. Fully Connected Layer

Fully connected layer (FC for short), the location of the fully connected layer is usually located in the later layers of the convolutional neural network, which is equivalent to the hidden layer in the traditional feedforward neural network and can be understood as a simple multiclassification neural network with the final output obtained by the Softmax function. If the number of neurons in the production before the fully connected layer is too large and the learning ability is high, it may lead to overfitting. Therefore, a dropout strategy can be introduced to randomly remove some neurons in the neural network to solve this problem.

### 3.2. Quantitative Model Design for International Trade

**Figure 4:**
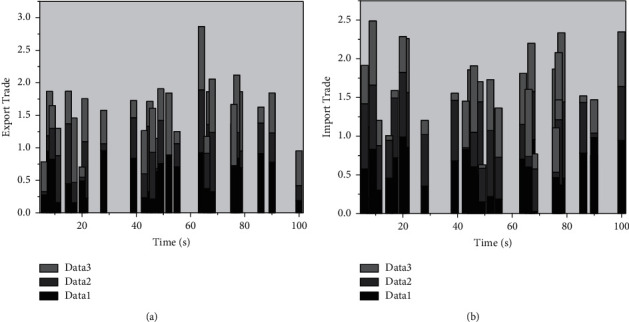
Impulse response of shocks to the import trade structure, export trade structure to the industrial structure.

In the broad sense, the structure of foreign trade consists of commodity structure, mode structure, pattern structure, and regional structure. In a narrow sense, foreign trade structure refers only to the design of foreign trade commodities, which refers to the share of certain types of things in international trade, including the construction of work in goods and the structure of business in services. The commodity structure can be divided into primary products and industrially manufactured goods according to the degree of processing, and the classification is only a highly generalized overall indicator. To compare China's merchandise trade structure with other BRICS countries more clearly, this section is based on the (SITC) developed by the United Nations, which classifies goods into ten major categories, including resource-intensive goods (SITC0-4), labor-intensive goods (SITC6, SITC8), and capital or technology-intensive goods (SITC5, SITC7). Resource-intensive goods are among the items included in primary products, and labor-intensive goods and capital or technology-intensive goods are among industrial manufacturers, which are industrial manufacturers divided according to the type of consuming factors. The impulse responses of shocks to the import trade structure and export trade structure in the industrial system are shown in Figure 4.

Spatial econometric theory indicates that the observations are not independent of each other. There are spatial correlation characteristics in the whole society, according to which spatial econometric models are used in this paper to process the sample data with spatial dependence characteristics. Spatial error models (SEM), spatial lag models (SLM), and spatial Durbin models (SDM) are commonly used in spatial econometric models. This paper chooses the spatial SDM model to analyze the impact of international trade and technological progress on energy efficiency. The SDM model explains economic problems by measuring direct, indirect, and aggregate effects, and the bias is likely to be small. The specific model form is

(5) $$\begin{array}{c} Y_{it}=\sum_{j=1}w_{ij}x_{it}+\sum_{j=1}w_{ij}y_{it}, \end{array}$$

where *Y*_*it*_ denotes total factor energy efficiency, *X*_*it*_ represents core explanatory variables, ∑_*j*=1_^*n*^*w*_*ij*_*x*_*it*_ and ∑_*j*=1_*w*_*ij*_*y*_*it*_ mean the spatial lagged terms of core explanatory variables and total factor energy efficiency, respectively, and *W* denotes the spatial weight matrix. The partial differential matrix decomposes the full effects to measure their direct and indirect effects. The spatial SDM model is further deformed into the following matrix form in this paper.

(6) $$\begin{array}{c} Y_{T}=\frac{\partial W+1}{\sqrt{\theta X_{T}-1}}+{\left(\partial W-1\right)}^{2}. \end{array}$$

The matrix form of equation (6) indicates that the mean values of the main diagonal elements of the matrix represent the changes in the energy efficiency of this unit caused by the changes in each explanatory variable, i.e., the direct effect and the mean values of the nonmain diagonal elements represent the changes in the energy efficiency of other units caused by the changes in each explanatory variable, i.e., the indirect effect (spatial spillover effect). The spatial weight matrix is essential for the regression estimation results. This paper draws on the existing literature to quantify the spatial relationships among the influencing factors in a matrix to accurately portray the relationships between the explanatory and explained variables. This paper constructs a 0-1 weight matrix for empirical study and selects an economic distance matrix and geographic distance matrix for robustness check. The contribution of import trade structure and export trade structure to the industrial design gradually increases over time. The gift of export trade change to the industrial structure change also gradually increases and stabilizes at about 17%. The contribution of import trade structure is significantly smaller than that of export trade, and the gift of import trade change to industrial structure change starts to rise after the second period. However, the contribution is still not high, stabilizing at less than 9%. This is also related to China's imports from BRICS countries to raw materials, energy, and other resource-based products, without sound effects to promote the development of similar related industries. The contribution of the scale of import and export trade with BRICS countries can only explain 26% of the industrial structure, indicating other factors with the higher assistance in industrial structure upgrading.

## 4. Analysis of Results

### 4.1. Deep Neural Network Model Analysis

**Figure 5:**
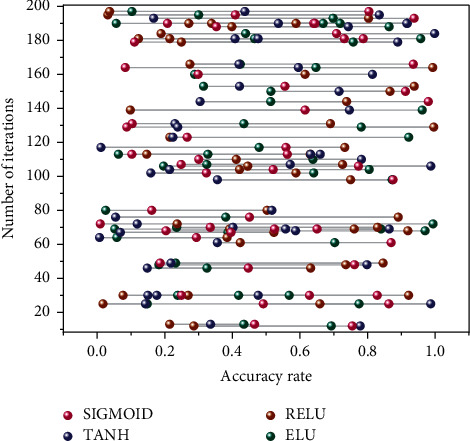
Variation of the accuracy rate with an increasing number of iterations.

The more complex the model structure of a convolutional neural network, the better its network performance will be compared, but this will also cause an increase in the number of parameters and computation. Then such a network model requires sufficient computer resources for processing, such as a high-performance graphics computing chip and more significant memory [25]. The larger network models may run slower or appear unworkable when limited computational resources and performance. The network can be compressed to accommodate computer devices to address the above problems. Still, suppose compressing the trained network reduces the number of parameters. In that case, the process is not only tedious but also may not improve the correctness of the web, so this chapter adopts a shallow approach to model simplification directly. The improved external network model is based on the structural framework of Alex Net and then combined with the simple idea to improve and optimize the structure, which can guarantee a faster and more accurate network model while training in a standard configuration computer. A simple record of a multibranch shallow network, although the number of parameters and accuracy is still not as good as Dense Net, it does consist of only a few layers of convolutional networks. The activation function is a nonlinear function that allows the model to fit high-dimensional data better. Four sets of experiments were conducted to compare the effects of activation functions on sports image classification results using the commonly used activation functions Sigmoid, Tanh, Re LU, and ELU. The changes in the accuracy of the four activation functions with an increasing number of iterations are shown in Figure 5.

**Figure 6:**
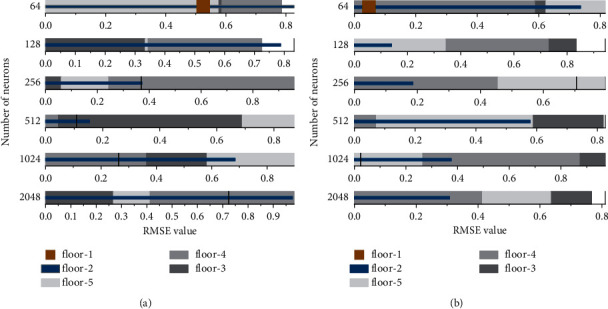
Performance of different numbers of neurons with the number of layers of hidden layers on two metrics.

The fully connected network settings in the CNN Decoder part of the model have a crucial impact on the final trading results, including the number of neurons and hidden layers. Considering the complex model construction in this paper, it will significantly increase the computational cost of the model if all the parameter combinations are taken into account to train the model. Therefore, several frequently used hyperparameter values are selected to coach the learning model in this paper. The parameters with the best model performance after use are chosen by experimental comparison. The experiments compare the transaction prediction models with the number of neurons in the implicit layer of the fully connected network of 64, 128, 256, 512, 1024, and 2048, respectively, and make the number of implicit layers of the transaction amount prediction model increase in steps of 1 starting from layer 1. In the validation set, the performance comparison of the experiments on the number of neurons and the number of hidden layers in this set of fully connected network settings is shown in Figure 6. The RMSE and MAPE metrics of the model perform better when the number of neurons of the fully connected network is 256, 512, and 1024; The number of neurons increases and the model complexity improves; the number of fully connected layers deepens, and the model nonlinear expression ability improves. All of them can theoretically improve the learning ability of the model. Therefore, in this paper, we choose to use a fully connected layer network structure with 512 neurons to optimize the performance and efficiency of the model. When the number of neurons in the fully connected network is 512, it can be seen from the above graph that the RMSE and MAPE metrics of the model perform poorly when the number of hidden layers is 1 and 2. However, as the network deepens, the model indicators decrease and then increase. This indicates that as the number of hidden layers increases, the fitting ability of the model will gradually increase. When the number of hidden layers increases beyond the optimal number of layers, the model may be overfitted, i.e., the fit to the case data is too fine, resulting in the RMSE and MAPE values of the model showing a process of first getting better and then getting worse. In summary, the model in this paper selects a 3-layer implicit layer containing 512 neurons per layer as the fully connected network in the penalty prediction model.

Suppose the network model needs to be used in some mobile or embedded devices for practical applications and better classification accuracy. In that case, it needs to be lightweight, that is, to improve the efficiency of the network. On the one hand, the more profound and complex the network model is the higher its accuracy, which will also cause many weights and parameters. It needs a good performance and a large storage device to save these parameters. The weight matrix is used for empirical study, and the economic distance matrix and geographical distance matrix are selected for robustness testing.

On the other hand, if the algorithm model takes too long to process, it will be less likely to be considered for use and affect the user's experience if used in practice. There are two ways to solve the efficiency problem: one is to use high-performance computer equipment; the other is to reduce the number of parameters and the amount of computation. For the improved shallow convolutional neural network, the most important thing is that the network model can be trained faster in a small computer device with low configuration to ensure good classification accuracy, so to improve the efficiency of the network, we need to consider the number of parameters and the amount of computation of the network model. The number of params is related to how much memory space the model needs to occupy, generally using 32 bit floating-point binary storage. Convolution simulates the response of a single neuron to a visual stimulus, and each convolutional neuron processes only data within the receptive field, i.e., local connectivity, which significantly reduces the number of parameters and improves the generalization ability of the model. The number of params of CNN mainly considers the parameters of convolutional and fully connected layers. The number of parameters of the CNN especially finds the parameters of the convolutional layers and fully connected layers. The computational volume is related to the processing speed of the algorithm, and its size is measured by theoretical floating-point operations (FLOPs). In this paper, only the convolutional and fully connected layers are considered to calculate the number of network parameters and computation, and other operations such as activation functions and BNs are ignored.

### 4.2. Quantitative Model Implementation of International Trade

**Figure 7:**
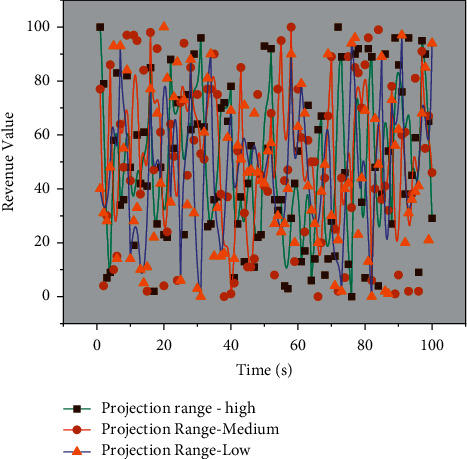
Projected range of revenue results for data provided.

The regression is performed using ordinary least squares (OLS). The corresponding AR terms are added to eliminate autocorrelation effects to obtain the fitted results. The industrial structure upgrading index is the dependent variable for the models with autocorrelation problems. The estimation results of the parameters are analyzed as follows: in terms of imports, resource-intensive products are significant at the 5% significance level with a coefficient of 0.095, indicating that increasing imports of resource-intensive products can promote industrial structure upgrading. While the import coefficients of labor-intensive products and capital- and technology-intensive effects do not pass the significance test, indicating that neither of these two product imports affects industrial structure upgrading. Imports from other BRICS countries are mainly resource-intensive products, and the trade volume far exceeds that of industrial manufacturers. By importing many agricultural products, energy minerals, and other raw materials from Brazil and Russia as industrial inputs, China can compensate for the lack of resources per capita and accelerate the rapid development of processing manufacturing industries. In addition, the small proportion of imported industrial manufactured goods, coupled with the limitations for the overall high-tech development capacity of BRICS member countries, the technology spillover effect is not prominent, thus making it difficult to impact industrial structure upgrading significantly. In terms of exports, labor-intensive products and capital-technology products pass the significance test at a 5% significance level with coefficients of 0.074 and 0.117, respectively, indicating that the expansion of exports of industrially manufactured goods contributes to the upgrading of industrial structure. The expected range of income results for the provided data is shown in Figure 7.

**Figure 8:**
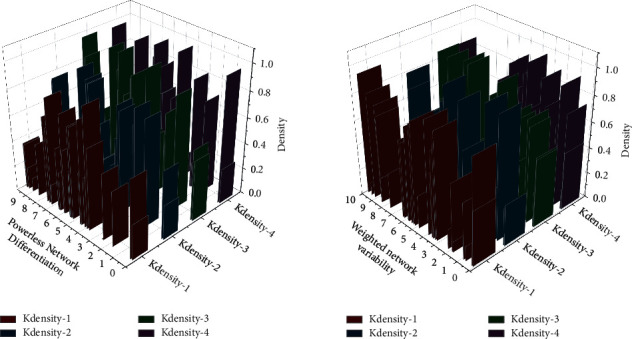
Comparison of quantitative models of international trade.

Since a double logit model is used, the coefficients represent elasticities, which means that every 1% increase in the export value of labor-intensive products will lead to a 0.074% increase in the industrial structure upgrading index; every 1% increase in the export value of capital- and technology-intensive products will cause a 0.117% increase in the industrial structure upgrading index. The product supply and demand markets of BRICS member countries have strong complementarity. Industries with excess capacity but still have comparative advantages or have entered maturity may be lacking resources for other BRICS countries, and demand and industrial structure upgrading can be effectively promoted through overall industrial transfer. There are various forms of spatial econometric models, and model selection is the first step of empirical research and an essential guarantee that the results of this paper are reasonable and accurate. This paper uses STATA software to conduct the Hausman test, Wald test, and LR test for spatial panel model selection. The *p* value corresponding to the Hausman test is 0.001, indicating that this paper should choose a more suitable fixed-effects spatial panel model. The *p* values corresponding to the Wald and LR tests are 0.001, suggesting that this paper determines the spatial SDM model more suitable. Therefore, this paper finally selects the fixed-effects spatial SDM model for empirical analysis. A comparison of the quantitative model of international trade is shown in Figure 8.

Import trade's direct and total effects on energy efficiency are significantly adverse at a 1% significance level, −0.075 and −0.128, respectively. The indirect impact is very harmful at a 10% significance level, −0.054. It indicates that overall, every 1% increase in the level of import trade leads to a significant decrease in energy efficiency by 0.128%, which causes a substantial reduction in energy efficiency in the home country by 0.075% and 0.054% in neighboring countries with a robust spatial spillover effect. This deviation from the expected result of this paper is consistent with the actual situation, and the reason for this phenomenon may be the “race to the bottom effect” and “negative self-selection effect” brought by import trade. To maintain their advantages, enterprises have to cut back on investment in research and development of energy-saving and emission reduction technologies and use low-priced and energy-consuming equipment and technologies for production. At the same time, it may also be because some importing countries are treated as “pollution sanctuaries,” that is, a country's low economic level and other reasons caused by their introduction of poor quality. Many reasons for the import trade are not conducive to energy efficiency improvement. The indirect and total effects of export trade on energy efficiency are positive at a 1% significance level, 0.105 and 0.126, respectively, while the direct impact is 0.021 but not significant. It shows that overall, every 0.01 increase in the level of export trade will lead to a substantial increase in energy efficiency by 0.021%, which will lead to a 0.126% increase in energy efficiency in the country and a significant 0.105% increase in energy efficiency in neighboring countries with a robust spatial spillover effect. This indicates that the improvement of export trade improves energy efficiency in the country and adjacent regions. Nowadays, the international market has higher and higher requirements for the environmental quality of traded products. Countries can no longer rely solely on the advantage of low prices to make profits in the international market. Hence, countries have to increase their investment in research and development of high-tech environmental technology to produce products that meet the global market's needs, i.e., the reverse technology spillover from export trade. Export trade's reverse technology spillover, competition, and industry linkage effects lead to energy efficiency improvements in their own countries and neighboring regions.

## 5. Conclusion

With the advancement of science and technology and the development of artificial intelligence and deep learning, the research on international trade has entered a new stage. This paper draws on prediction models widely used in various fields to construct deep neural network (DNN) models with multiple implicit layers and use the models to forecast trade. By comparing a deep neural network with multiple hidden layers to a BP neural network with one hidden layer, the results show that a neural network with multiple hidden layers can learn features that can portray the essential properties of the data and further improve the prediction performance of the model. The deep neural network can effectively overcome the difficulty of training deep neural networks by using a “layer-by-layer initialization” strategy. The deep neural network can effectively overcome the problem of training deep neural networks and reduce the complexity of model implementation. This paper constructs a quantitative trade model based on the Melitz-Chaney model with multisector extensions and heterogeneous enterprise trade theory. Compared with previous quantitative trade studies, the modeling approach applied in this paper is more standardized, structured, and replicable. This model can be combined with trade data to quantitatively analyze issues in international trade, especially those related to trade policy. Using a comparative static research approach, we have simplified the set of general equilibrium equations used for numerical simulations, eliminating exogenous variables that are difficult to identify and estimate, making applying the model for counterfactual studies much less complicated.

## Data Availability

The data used to support the findings of this study are included within the article.
